# Whey-Derived Peptides at the Heart of the COVID-19 Pandemic

**DOI:** 10.3390/ijms222111662

**Published:** 2021-10-28

**Authors:** Yara Chamata, Kim G. Jackson, Kimberly A. Watson, Paula Jauregi

**Affiliations:** 1 Harry Nursten Building, Department of Food and Nutritional Sciences, University of Reading, Reading RG6 6DZ, UK; k.g.jackson@reading.ac.uk (K.G.J.); pjauregi@azti.es (P.J.); 2 Health and Life Sciences Building, School of Biological Sciences, University of Reading, Reading RG6 6EX, UK; k.a.watson@reading.ac.uk

**Keywords:** whey peptides, molecular docking, hypertension, ACE2, COVID-19, ACE inhibitory activity, renin–angiotensin system

## Abstract

The renin–angiotensin system (RAS) is a key regulator of blood pressure and hypertension. Angiotensin-converting enzyme 2 (ACE2) and angiotensin-converting enzyme I (ACE) are two main components of the RAS that play a major role in blood pressure homeostasis. The severe acute respiratory syndrome coronavirus 2 (SARS-CoV-2) uses ACE2 as a receptor to enter cells. Despite some controversies, numerous studies have reported a significant association between the use of ACE inhibitors and reduced risk of COVID-19. In our previous studies, we produced and identified peptide sequences present in whey hydrolysates exhibiting high ACE inhibitory activity. Therefore, the aim of this work is to obtain an improved understanding of the function of these natural peptides as RAS inhibitors and investigate their potential therapeutic role in the COVID-19 pandemic. The molecular interactions between peptides IPP, LIVTQ, IIAE, LVYPFP, and human ACE2 were assessed by employing a molecular docking approach. The results show that natural whey-derived peptides have a dual inhibitory action against both ACE and ACE2. This dual activity distinguishes these ACE inhibitory peptides from synthetic drugs, such as Captopril and Lisinopril which were not shown to inhibit ACE2 activity, and may represent a potential strategy in the treatment of COVID-19.

## 1. Introduction

Cardiovascular diseases (CVDs) are reported to be the leading cause of death globally [1]. Elevated blood pressure (hypertension) is one of the most important and well-defined modifiable risk factors for the development of CVDs [2]. Thus, the effective control of blood pressure plays a key role in preventing hypertension-related deaths [3,4].

### 1.1. The Classical and Counter-Regulatory Renin–Angiotensin System (RAS) Pathways

**Figure 1:**
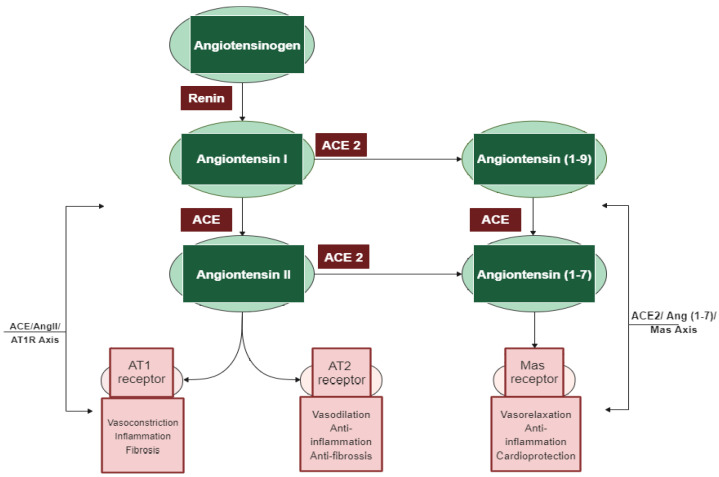
The two main axes of the renin–angiotensin system (RAS) cascade and their opposing effects. Adapted from D’ardes et al. [23].

Among the different regulatory and contra-regulatory systems contributing to the pathogenesis of cardiovascular and renal diseases, the renin angiotensin system (RAS) is one of the main therapeutic targets for CVDs as well as a key player that regulates blood pressure. The RAS pathway includes a cascade of proteases generating some bioactive molecules (Figure 1) [5]. Renin is a glycoproteolytic enzyme secreted by the juxtaglomerular cells of the afferent arterioles of the kidney. The liver-derived angiotensinogen acts as the substrate for renin, which cleaves angiotensinogen to form the decapeptide angiotensin I (Ang I) [6]. Angiotensin-converting enzyme I (ACE; dipeptidyl carboxypeptidase, EC 3.4.15.1) is a zinc metallopeptidase that is found in male genitals and in vascular endothelial, neuro-epithelial, and absorptive epithelial cells [7,8,9]. It displays both endopeptidase and exopeptidase activities, acting on a wide range of substrates [10]. ACE hydrolyzes the inactive decapeptide Ang I into the strong vasoconstrictor angiotensin II (Ang II). Additionally, ACE promotes the inactivation and degradation of the catalytic function of vasodilator bradykinin (BK) into inactive BK (1–7) and BK (1–5) [11,12,13]. By promoting the production of the potent vasoconstrictor Ang II, as well as by degrading the vasodilator BK, ACE plays a dual role in the RAS. In this respect, Ang II is a key component of the RAS pathway, exerting its effects via two G protein-coupled receptors, namely angiotensin type 1 (AT1) and type 2 (AT2). Most of the pathophysiological effects of Ang II are mediated through AT1 receptors, leading to vasoconstriction, cardiovascular inflammation, and aldosterone secretion [14]. The AT2 receptor is associated with effects that counteract those of the AT1 receptor; however, many functions of the AT2 receptor are less clear and studies reporting its importance are controversial [15].

In addition to the classical components of the RAS pathway (renin, ACE, Ang II, AT1, and AT2 receptors), novel peptides such as angiotensin 1–7 (Ang 1–7) and receptors such as angiotensin-converting enzyme 2 (ACE2) appear to play a central role in the regulation of the system [16]. ACE2 is an 805 amino-acid Type-I transmembrane protein that functions as a zinc metalloenzyme and monocarboxypeptidase [17]. Its extracellular domain consists of a single catalytic metallopeptidase that shares 61% sequence similarity and 42% sequence identity with the catalytic domain of ACE. ACE2 is active and expressed in most tissues; however, the highest expression of ACE2 is mainly observed in vascular endothelial cells and in the renal tubular epithelium [18]. Ang II appears to be the major substrate for ACE2 [18,19]. Ang (1–7) is a vasodilator with anti-proliferative effects produced by the catalytic activity of ACE and ACE2 from Ang I or Ang II [16]. The biologically active peptide Ang (1–9) is formed through the hydrolysis of the amino acid leucine from the C-terminal of Ang I by ACE2 (Figure 1) [18]. Ang (1–9) is subsequently cleaved by ACE and the neutral endopeptidase 24.11 (NEP) to generate Ang (1–7) [19], which can also be generated directly through the cleavage of the amino acid phenylalanine at the C-terminal of Ang II [20]. ACE2 therefore plays a key role as a regulator of the RAS pathway through degrading the vasoconstrictor/proliferative peptide Ang II and producing the vasodilator/antiproliferative peptide Ang (1–7) [13]. Additionally, the identification of the G protein-coupled receptor Mas, as a receptor of Ang (1–7), was another pivotal step to establish the relevance of Ang (1–7) [21]. The ACE2/Ang (1–7)/Mas axis is now accepted to counteract most of the harmful actions of the ACE/Ang II/AT1 receptor axis [22]. ACE2 is thus a key counter regulatory enzyme and a potent modulator of blood pressure [23].

The discovery of these novel components (ACE2, Ang (1–7), and Mas receptor), which have been recently added to the RAS system, has completely altered our understanding of the regulatory mechanisms of this pathway. It is now widely accepted that the system is dual and consists of two axes: the primarily deleterious axis consisting of ACE/Ang II/AT1 and the beneficial axis consisting of ACE2/Ang-(1–7)/Mas. These novel RAS elements thus open up new opportunities for interfering with the activity of the system and invigorating the development of new cardiovascular drugs targeting the beneficial and counter-regulatory axis of the RAS [24].

### 1.2. Characteristics of SARS-CoV-2

The novel coronavirus (COVID-19) is a pandemic that emerged in late 2019 and arguably became one of the greatest public health challenges of our time. COVID-19 is continuing to spread around the world, with the World Health Organization reporting more than 240 million confirmed cases and 4 million deaths globally at the time of writing this article (October 2021) [25].

Coronaviruses belong to the subfamily Coronavirinae in the family Coronaviridae of the order Nidovirales (International Committee on Taxonomy of Viruses) [26]. The subfamily Coronavirinae can be divided into four genera: alpha, beta, gamma, and deltacoronavirus [27]. According to Zhu et al. [28], the severe acute respiratory syndrome coronavirus 2 (SARS-CoV-2) belongs to the betacoronavirus genus. The alpha and betacoronaviruses are mainly associated with infections in mammals, with severe acute respiratory syndrome (SARS) coronavirus (SARS-CoV), and with Middle East respiratory syndrome (MERS) coronavirus (MERS-CoV), representing two prominent examples of highly pathogenic coronaviruses causing severe respiratory disease in humans. Due to the greater resemblance of the novel virus with SARS-CoV, the Coronavirus Study Group (CSG) of the International Committee on Taxonomy of Viruses (ICTV) named it “SARS-CoV-2” [26]. Genome-wide phylogenetic analysis shows that SARS-CoV-2 shares 50% and 79.5% sequence identity to MERS-CoV and SARS-CoV, respectively [28,29]. Similar to other betacoronaviruses, SARS-CoV-2 contains a positive-sense, single-stranded RNA genome of 29.9 kb in size (National Microbiology Data Center) [30], encoding structural proteins which include the spike (S), envelope (E), nucleocapsid (N), and membrane (M) [31]. Besides the genes encoding structural proteins, other genes encode non-structural proteins crucial for virus replication and translation of the viral genome in the host cells [32,33].

ACE2 is a type I membrane protein. It consists of the C-terminal collectrin-like domain (CLD) and the N-terminal peptidase domain (PD) that provide direct binding sites for the coronavirus S protein (Yan et al., 2020). According to Wrapp et al. [34], SARS-CoV-2 binds to ACE2 with much higher affinity (about 10–20 times higher) than the binding of the SARS-CoV S protein to ACE2, which explains why SARS-CoV-2 is highly infectious. ACE2 is highly expressed in the mouth and tongue, which facilitates viral entry for SARS-CoV-2. It is also expressed highly throughout the gut [35] and in the lower lungs on alveolar epithelial cells type I and type II [34]. Post-infection, the S protein expressed on the viral envelope attaches itself to ACE2 on the alveolar surface. This binding stimulates the clathrin-dependent endocytosis of the SARS-CoV-2 and ACE2 complex, which includes fusion at the cell membrane. Once SARS-CoV-2 is inside the cells, it exploits the alveolar cells’ endogenous transcriptional machinery to replicate and spread through the entire lung [36].

### 1.3. Controversies Regarding the Role of ACE2 in COVID-19

Besides its role as a SARS-CoV-2 receptor, ACE2 is established for its role in hypertension by negatively regulating the RAS through modulating blood pressure to maintain blood pressure homeostasis. The unique interaction of SARS-CoV-2 and host cell receptor ACE2 provides a critical link between COVID-19, hypertension, and CVD [37,38]. As ACE2 has been identified as the crucial receptor for SARS-CoV-2, the entire RAS should be evaluated when addressing the COVID-19 pandemic. According to Sriram and Insel [39], the imbalance in the action of ACE2 and ACE is one of the main culprits of COVID-19 pathobiology. Hence, in order to treat ACE-2-mediated COVID-19, there are two primary approaches suggested to restore ACE/ACE2 balance in the literature: (i) ACE inhibitors/increasing ACE2 or Ang (1–7) levels, and/or (ii) seeking new molecules targeting the S protein or ACE2 receptor to prevent infection by SARS-CoV-2 [40,41,42].

From a therapeutic perspective, activating the ACE2/Ang (1–7)/Mas axis or inhibiting the ACE/Ang II/AT1R axis could be promising [36,43,44]. Previous research has demonstrated that SARS-CoV infection significantly decreases ACE2 levels in infected mice [45]. The binding of SARS-CoV-2 to ACE2 also reduces levels of ACE2, thereby inhibiting the ACE2/Ang (1–7) pathway and shifting the balance of the RAS system, consequently leading to the exacerbation of acute severe pneumonia [46]. By inhibiting the conversion of Ang I to Ang II, ACE inhibitors reduce Ang II production and subsequently the effects are triggered by its interaction with the receptor AT1R, namely vasoconstriction [23]. The hypothetical association between treatment with ACE inhibitors and severe COVID-19 disease has been intensely debated in the literature [47,48,49,50]. One hypothesis suggests that the use of these drugs could be harmful in the sense that increased expression of ACE2 receptors may enhance viral-binding and entry [51,52]. The other hypothesis proposes that ACE inhibitors could be protective by decreasing the production of Ang II and boosting the production of Ang (1–7), which attenuates inflammation and fibrosis, and hence could attenuate lung injury [53,54]. Various large cohort studies have suggested that the use of ACE inhibitors was not correlated with increased SARS-CoV-2 infection but was in fact linked to a reduced risk of mortality in hospitalized COVID-19 patients [55,56,57].

### 1.4. Whey-Derived Peptides as Promising Therapeutic Candidates

Ever since the pandemic brought chaos to lives across the globe, scientists have been making extraordinary efforts to explore therapeutic candidates, such as developing effective vaccines and drugs against COVID-19, to reduce the severity of the outbreak. Given the significance of the ACE2 receptor, research groups have been seeking new molecules targeting this receptor to prevent infection by SARS-CoV-2 and mitigate the development of COVID-19 disease [38,56,58]. Many of the recent studies have investigated the potential of chemical compounds such as flavonols [59] and peptides as novel therapeutic inhibitors against SARS-CoV-2, targeting the ACE2 receptor [40,41,42]. Peptide and peptide-based inhibitors represent attractive candidates that hold great promise for the development of ACE2 inhibitors due to their safety, specificity, and efficacy compared to small molecule drugs. Antiviral peptides can also be rationally designed and optimized based on the known structures of viral proteins, as these can be developed to be highly specific for their respective targets [60,61]. Strategies to interfere with the interaction of the S protein with the ACE2 receptor have been previously examined with SARS-CoV [62]. Hence, antagonist ACE2 proteins or their derived peptides may not only be a treatment for preventing the spread of SARS-CoV-2 but also for the modulation of the RAS. These proteins and the derived peptides could be used both to protect patients with COVID-19 disease and to limit the spread of the current SARS-CoV-2 and other coronaviruses by preventing the replication of the virus and development of SARS in the lung [61].

Computational approaches play a considerable role in the process of rapid drug development and discovery in a cost and time-efficient manner. In the literature, many researchers have aimed to identify novel ACE2 inhibitors utilizing a molecular docking strategy [40,41,42].

Given the high sequence similarity and sequence identity between ACE and ACE2 [18], and the reported reduced risk of mortality and disease associated with use of ACE inhibitors among COVID-19 patients [55,56,57], investigating the ability of ACE inhibitors to block ACE2 interaction with the SARS-CoV-2 S protein would be promising. Various animal and plant proteins have been used in the development of functional foods providing ACE inhibitory activity; however, milk is the main source of antihypertensive ACE-inhibitory peptides reported to date [63,64]. In fact, previous studies have demonstrated that milk-derived peptides are associated with lower blood pressure [65], with some researchers generating evidence to support the beneficial impact of milk proteins on vascular health [66].

In our previous work, we characterized ACE inhibitory peptides produced by enzymatic hydrolysis of whey proteins [67]. Peptide sequences were identified as major peptides in fractions from the enzymatic hydrolysates CDP (casein-derived peptides) and β-lactoglobulin. The well-known antihypertensive peptide Ile-Pro-Pro (IPP), along with some other novel peptide sequences that have structural similarities with the reported ACE inhibitory peptides, such as Leu-Val-Tyr-Pro-Phe-Pro (LVYPFP), Leu-Ile-Val-Thr-Gln (LIVTQ), and Ile-Ile-Ala-Glu (IIAE), was characterized and identified by a combination of chemical characterization (LC/MS; MS/MS) and structure-activity relationship data. These peptides produced naturally from whey by enzymatic hydrolysis interacted with residues of human ACE, in common with potent ACE-inhibitory drugs, such as Sampatrilat, Captopril, Lisinopril, and Elanapril, which suggests that these natural peptides may be potent ACE inhibitors [67,68]. The present study aims to explore the efficacy of a natural therapeutic strategy that targets both RAS axes for potential treatment and/or prevention of COVID-19. Herein, we investigate the potential interactions between whey protein-derived peptides with high ACE inhibitory activity (IPP, IIAE, LIVTQ, and LVYPFP) and human ACE2, employing a molecular docking approach. The overall aim is to obtain an improved understanding of such peptides’ function as RAS inhibitors and to assess their potential therapeutic role at the heart of the COVID-19 pandemic.

## 2. Results and Discussion

### 2.1. Molecular Docking

In this study, molecular docking was conducted to elicit the potential molecular interactions between the specific amino acids at the binding site of human ACE2 and our previously identified whey protein-derived peptide sequences with high ACE inhibitory activities.

The peptide sequences were docked into the binding site of human ACE2 using the X-ray crystallographic structure of the human ACE2 receptor (PDB code 6M0J). As 6M0J does not contain a co-crystallized ligand, to validate our docking approach, we used the co-crystallized MLN-4760 ACE2 receptor complex (PDB code 1R4L), whereby we extracted the co-crystallized ligand MLN-4760 and re-docked it into the prepared protein 1R4L. The root-mean square deviation (RMSD) between the docked conformation (as generated by superimposition in the program PyMol) and the native co-crystallized ligand conformation was 0.3 Å, which is well within the 2 Å grid spacing used in the docking procedure, demonstrating that the docking method used was valid and reliable. Furthermore, the interactions between the docked ligand and the prepared target receptor mimicked those observed in the crystal structure of the same protein (PDB code 1R4L) [69].

**Figure 2:**
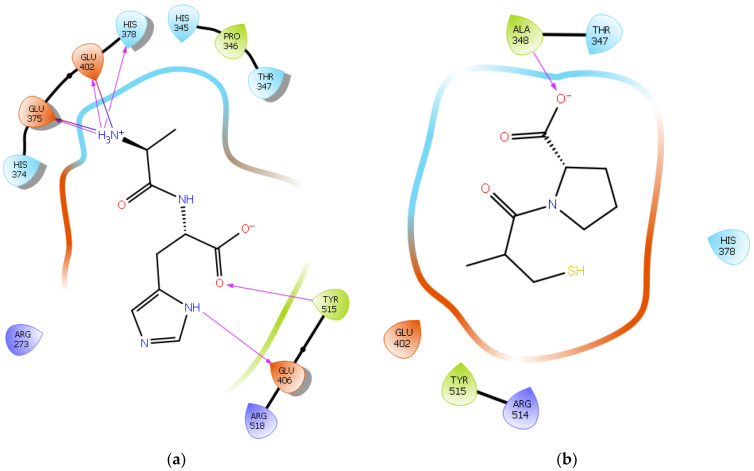
(**a**) Docking results of the peptide Carnosine in the human angiotensin-converting enzyme 2 (ACE2) active site. The interactions of human ACE2 residues with Carnosine (represented in black) are indicated by arrows of different colors, with purple representing hydrogen bond interactions and blue arrows representing salt bridge interactions. (**b**) Docking results of the synthetic drug Captopril in the human angiotensin-converting enzyme 2 (ACE2) active site. The interaction of human ACE2 residues with Captopril (represented in black) is indicated by a purple arrow representing hydrogen bond interactions.

To further validate our method, the ligand Carnosine was docked into the prepared X-ray crystal structure of the human ACE2 receptor (PDB code 6M0J) to be used for subsequent docking runs. As Q9BYF1 is the UniPROT code for both 6M0J and 2AJF structures and given that these are 100% identical ACE2 sequences in both X-ray crystal structures (Figures S1–S3), according to the EMBOSS needle results (Figure S4), the interactions between the docked ligand Carnosine and those observed in the crystal structure (PDB code 2AJF) were compared (Figure 2a and Figure S5) [40]. In our docking study, Carnosine interacted with key amino acid residues, namely Glu 375, His 378, Glu 402, and Tyr 515 in the active site of ACE2, in accordance with observations reported in the literature [40,69,70,71]. In a study providing structural insights for the differences in the inhibition pattern and substrate specificity for ACE and ACE2, amino acid residues His 374, His 378, Glu 375, Tyr 515, Glu 402, and Glu 406 were characterized in the active site of ACE2 [70]. These observations were corroborated in the first reported crystal structure of ACE2 in its native and inhibitor-bound states, where key binding residues His 374, His 378, and Glu 402 were identified [69]. According to Saadah et al. [40], Carnosine interacted with amino acid residues His 378, Glu 402, and Tyr 515 at the active site of ACE2, which was also confirmed in our docking approach.

The synthetic ACE inhibitory drug Captopril was also docked into 6M0J. According to the docking results, Captopril did not interact with any key binding residues in the ac-tive site of ACE2 and formed only one potential hydrogen bond with the backbone of the amino acid residue Ala 348 (Figure 2b and Figure S6). These observations are in line with those reported in other studies, wherein ACE inhibitors such as Captopril could not inhibit ACE2 [70,72].

Following validation, the human ACE2 receptor (PDB code 6M0J) was then used as the target molecule for docking the peptide sequences of interest into its active site.

**Figure 3:**
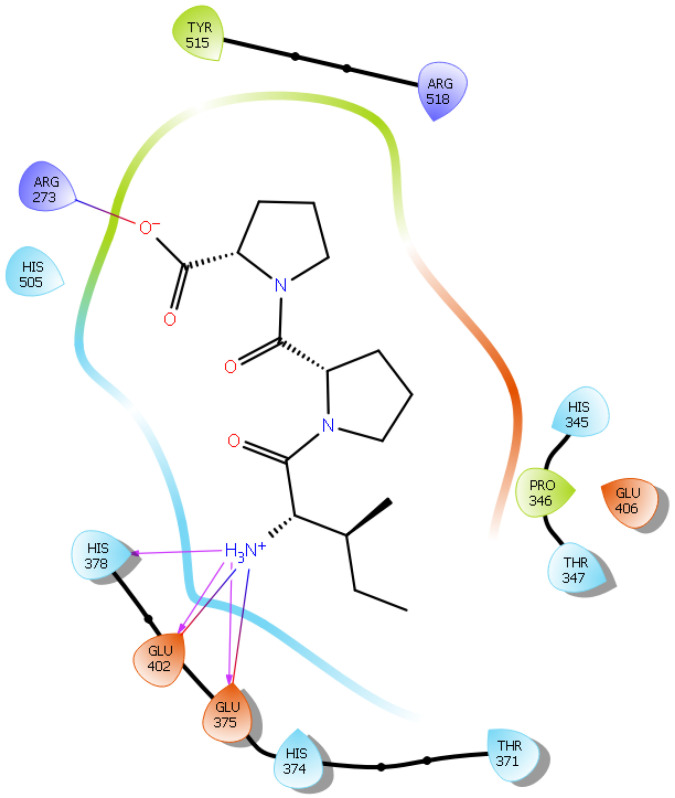
Docking results of the peptide IPP in the active site of the human angiotensin 2-converting enzyme (ACE2). Interactions of human ACE2 residues with the peptide IPP (represented in black) are indicated by arrows of different colors, with purple arrows representing hydrogen bond interactions and blue arrows representing salt bridge interactions.

**Figure 4:**
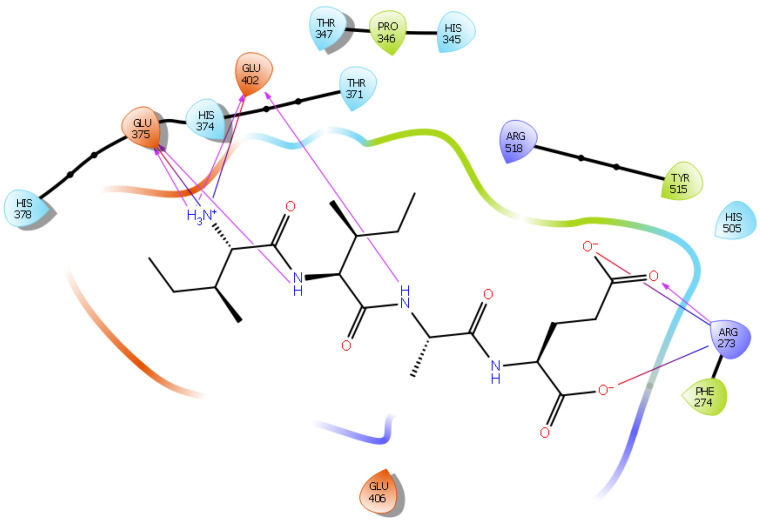
Docking results of the peptide IIAE in the active site of human ACE2. The interactions of human ACE2 residues with the peptide IIAE (represented in black) are indicated by arrows of different colors, with purple arrows representing hydrogen bond interactions and blue arrows representing salt bridge interactions.

**Figure 5:**
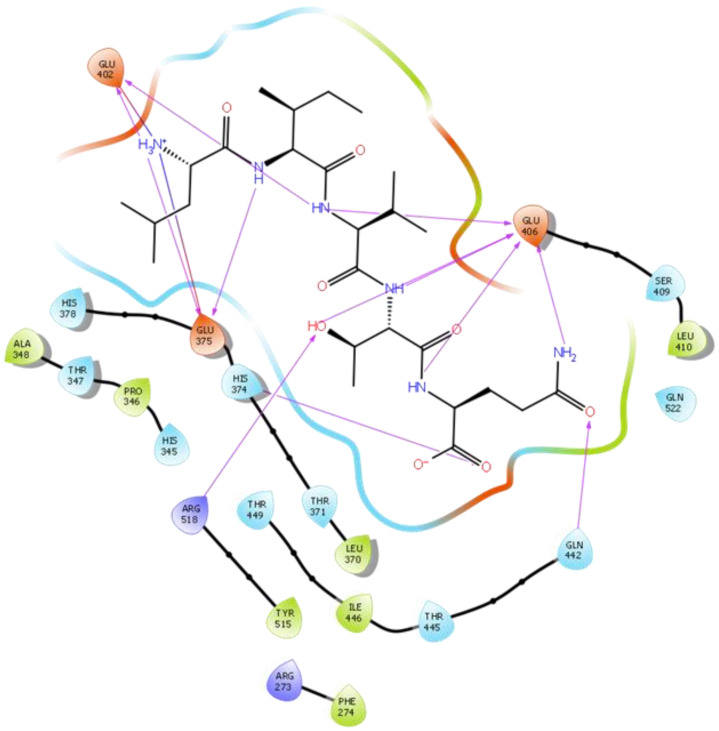
Docking results of the peptide LIVTQ in the human ACE2 active site. The interactions of human ACE2 residues with the peptide LIVTQ (represented in black) are indicated by arrows of different colors, with purple arrows representing hydrogen bond interactions and blue arrows representing salt bridge interactions.

**Figure 6:**
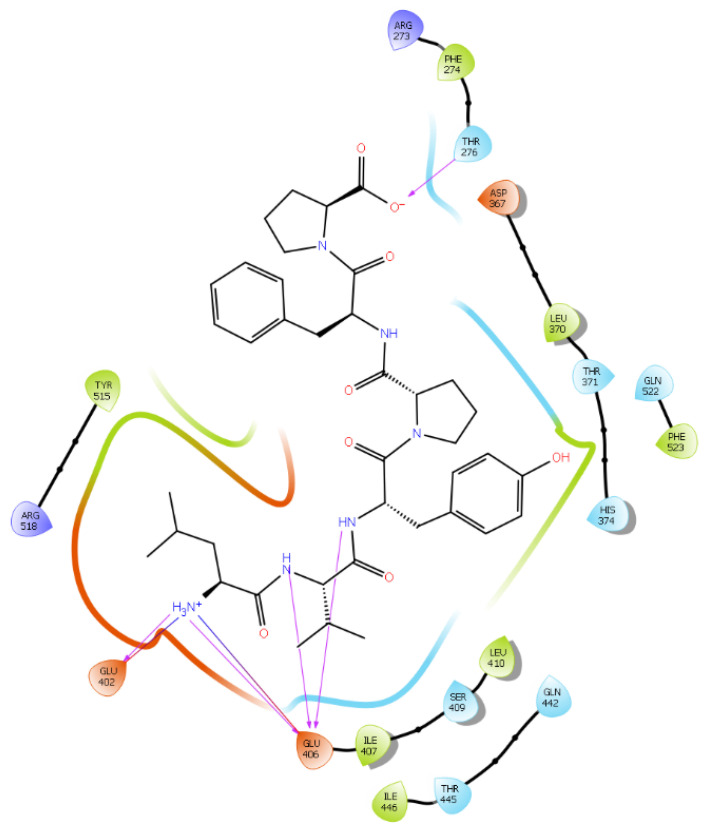
Docking results of the peptide LVYPFP in the human ACE2 active site. The interactions of human ACE2 residues with the peptide (represented in black) are indicated by arrows of different colors, with purple arrows representing hydrogen bond interactions and blue arrows representing salt bridge interactions.

Hydrogen bond interactions play a crucial role in the specificity and stability of protein–ligand interactions. The results of ligand-driven docking into the binding site of ACE2 are summarized in Figure 3, Figure 4, Figure 5 and Figure 6, and Figures S7–S10, and Table 1 and Table S1. It is known that His 374, His 378, and Glu 402 are important ligand-binding residues in the zinc-binding site of ACE2 [69,70,71,72]. In the current study, IPP showed potential interactions with the key residues His 378 and Glu 402 through hydrogen bond interactions at distances of 2.4 Å and 2.9 Å, respectively. Interestingly, IPP also interacted with these two amino acid residues, namely His 378 and Glu 402, similarly to Carnosine, the best-known drug candidate to match an ACE2 inhibitor structure [40]. Additionally, IPP formed a salt bridge and a hydrogen bond with amino acid residue Glu 375, another key active amino acid residue in ACE2 (Table 1, Figure 3) [70,71]. IIAE, LIVTQ, and LVYPFP also interacted with residue Glu 402 in common with the potent ACE2 inhibitor Carnosine. This was done via hydrogen bonding and salt bridge interaction at distances of 2.9 Å and 4.3 Å, respectively, for IIAE (Table 1, Figure 4); through two hydrogen bonds at distances of 2 Å and 2.6 Å, and through one salt bridge interaction at a distance of 4.3 Å for LIVTQ (Table 1, Figure 5); and through hydrogen bonding and salt bridge interactions at distances of 2.9 and 3.8 Å, respectively, for LVYPFP (Table 1, Figure 6). Additionally, IIAE formed two hydrogen bonds and one salt bridge interaction with key binding amino acid residue Glu 375 (Table 1, Figure 4) [70,71].

In another molecular docking study conducted by Upreti et al. [73], chloroquine phosphate, a commercial ACE2 inhibitor, exhibited well-established hydrogen bonds with amino acid residues Glu 406, Asp 367, Asp 269, and Phe 274. Peptides LIVTQ and LVYPFP also interacted with the amino acid residue Glu 406 through hydrogen bonds and salt bridge interactions (Table 1; Figure 5 and Figure 6). Peptide LIVTQ additionally interacted with key residues His 374 and Glu 375 at distances of 2.8 Å and 3.5 Å, respectively (Table 1 and Figure 5). Moreover, Arg 273 is a key amino acid residue for substrate-binding in ACE2 that was found to form a salt-bridge with the C-terminal of the potent and selective human ACE2 inhibitor MLN-4760 [69,71,72]. Both peptides IPP and IIAE formed salt bridge interactions and hydrogen bonds with amino acid residue Arg 273 (Table 1; Figure 3 and Figure 4).

### 2.2. ACE and ACE2

Sequence alignment of ACE2 with ACE revealed the high conservation between these two enzymes. An analysis of the critical active site residues between ACE and ACE2 demonstrated that these two proteins are structurally well conserved. Since their active site structures are highly conserved and there exists a strong similarity between the catalytic domains of ACE and ACE2, consequently, the catalytic mechanism of ACE2 closely resembles that of ACE. However, due to differences in substrate specificity, distinctive key differences are present between the active site pockets of ACE and ACE2. Indeed, these differences occur in the ligand-binding pockets, particularly in the binding of the peptide C-terminal and at the S2′ subsite. The cavity in ACE2 is smaller than that of ACE, which allows an extra amino acid to bind in the specificity pocket. Additionally, the S1 subsite of ACE is larger than that of ACE2 [70]. In our previous work, we provided strong in vitro evidence for the ACE inhibitory activity of peptide sequences IPP, IIAE, LIVTQ, and LVYPFP [67,68]. Although all four peptides have been found to exhibit high ACE inhibitory activity, peptide IIAE formed strong hydrogen bonds with the amino acid residues Gln 259 and Thr 358 in the active site of ACE, in common the ACE inhibitory drug Sampatrilat. IIAE also interacted with the amino acid residues Gln 259 and His 331, in common with other ACE-inhibitory drugs such as Captopril, Lisinopril, and Elanapril, and with the amino acid residue Asp 140, in common with Lisinopril. Additionally, IPP has been identified as the most potent ACE inhibitor from milk protein [74]. When compared to the other peptides’ interactions with ACE, IIAE and IPP seem to display the highest ACE inhibitory activities. Notably, according to the ACE2 docking results, both peptides IPP and IIAE also seem to display the highest ACE2 inhibitory activity.

### 2.3. Potential Use of ACE Inhibitors in the Treatment of COVID-19

To date, there is no effective drug available to treat COVID-19 patients [75]. Although COVID-19 vaccines were shown to be closely associated with a significant reduction in symptomatic infections [76], vaccine hesitancy is widespread worldwide, which could hinder populations from achieving herd immunity [77]. The rapid global emergence of novel SARS-CoV-2 variants, the unequal international distribution of COVID-19 vaccines, and slow vaccine rollouts, especially in developed countries, could also be significant factors obstructing the achievement of herd immunity and the end of the pandemic. Although antimalarial drugs chloroquine and hydroxychloroquine, and some synthetic drugs such as remdesivir [75] and ritonavir/lopinavir [75,78] are currently used to treat COVID-19 patients, currently, there remains no effective and approved drug available against COVID-19 [79]. Various side effects associated with the aforementioned drugs were also observed among treated patients [75,78], delaying widespread acceptance and administration.

Consequently, identifying safe and effective compounds that can restrain the entry of SARS-CoV-2 into host cells via ACE2 is a priority for the scientific community. In this respect, an active area of research is the impact of milk/whey-derived bioactive peptides and their potential health benefits as ingredients of health-promoting functional foods [80]. Peptide sequences from whey proteins exhibit different bioactivities, including ACE inhibitory activity. In fact, milk is the main source of antihypertensive ACE-inhibitory peptides reported to date [63,81].

In the scientific community, controversy has arisen regarding whether the use of ACE inhibitors would be harmful or beneficial in the context of the COVID-19 pandemic [48,49,50,82,83,84]. Although increased COVID-19 disease severity seems to manifest in people with cardiovascular comorbidities [37,85], it is suggested that this association could be related to advanced age and obesity [85]. Moreover, there seems to be growing evidence that the use of ACE inhibitors does not worsen the prognosis of COVID-19 [86]. In fact, in a cohort study including 8.3 million people, ACE inhibitors were not found to be significantly associated with increased risks of COVID-19 disease, nor of requiring ICU care [57]. In agreement, another meta-analysis study also reported that the use of ACE inhibitors was not associated with requiring intensive care, mechanical ventilation, progression to severe disease, and increased risk of death. However, some researchers have reported a 16% reduction in the risk of COVID-related mortality with the use of ACE inhibitors [83].

Some studies suggest that ACE inhibitors could even play a protective role in hypertensive patients by averting organ injury [87]. Indeed, in vivo models support the role of ACE inhibitors in blunting lung injury and exerting health benefits in both human and animal trials [88,89,90,91]. Data from human studies also revealed that ACE inhibitors can reduce or prevent pneumonia [92,93]; specifically, the (i) treatment of chronic obstructive pulmonary disease with ACE inhibitors was found to reduce disease complications and (ii) treatment with ACE inhibitors was shown to mitigate the effects of radiation pneumonitis [94]. In short, there is consistent evidence indicating that ACE inhibitors seem to have beneficial effects in modulating lung damage, including in the context of pulmonary injury caused by viral infection. Due to insufficient evidence of the potentially harmful effects of ACE inhibitors and considering the overwhelming evidence supporting their benefits, multiple scientific societies rejected the recommendation to discontinue ACE inhibitors in the context of the COVID-19 pandemic [95,96,97]. Interestingly, ACE inhibitors were reported to be associated with significant pulmonary inflammatory response reductions in patients admitted with viral pneumonia [98] and attenuated inflammatory response in COVID-19-infected patients [99,100]. This emerging evidence prompted many researchers to advocate for the use of RAS inhibitors in the therapeutic management of COVID-19 infection [83].

Regarding the role of the RAS pathway in the pathophysiology of COVID-19 and SARS-CoV-2 infection, there are two primary theories. First, data from the literature have shown that Ang II-mediated inflammation is a main mediator of acute lung injury and fibrosis [45,101,102]. Similar to SARS-CoV, loss of ACE2 activity and expression could lead to an increase in Ang II levels in the lungs and could consequently induce COVID-19 acute lung injury. One study reported significantly higher Ang II levels in COVID-19 patients that correlated with viral load and indicators of lung injury. However, this study had considerable methodological limits: only 12 patients took part in the clinical study and circulating levels of ACE and/or ACE2 were not determined [5,53,103]. Furthermore, data from the original SARS-CoV epidemic indicated that infection with SARS-CoV-2 may lead to ACE-2 dependent myocardium infection, which results in decreased cardiac ACE2 expression, accelerating acute heart injury [104]. However, it is important to note that there is no clinical data to confirm this.

Second, there is concern that ACE inhibitors may potentially increase the expression and levels of ACE2 in the lungs, which facilitates SARS-CoV-2 infection such that administering ACE inhibitors may increase the risk of severe and fatal disease [52,105]. In select animal models, ACE inhibitors were able to increase heart and kidney ACE2 expression [51,106]. However, there are no data proving that these compounds can increase lung ACE2 expression in both animal models and human trials. In a similar manner, there are no available data demonstrating that the increased expression of ACE2 would necessarily indicate an increased risk of disease severity or infection, or that the use of these agents is correlated with increased virulence or viral infectivity. In fact, there does not appear to be any consistent association between increased ACE2/Ang (1–7)/Mas pathway activity and expression, and the use of ACE inhibitors in the few clinical studies assessing the effect of ACE inhibitors on the ACE2/Ang (1–7) pathway [107,108,109,110]. Although there is a lack of evidence to demonstrate the effect of ACE inhibitors on ACE2 expression and thus SARS-CoV-2 infectivity, the bulk of the experimental evidence indicates that ACE inhibitors may reduce the action of Ang II and consequently attenuate Ang II-driven acute lung injury [53,54]. ACE inhibitors therefore offer promise as potential novel therapeutics to treat COVID-19 disease [46].

Intriguingly, based solely on experimental studies in which RAS inhibitors were administered in vivo [111,112,113,114,115], Zamai, 2020 [116] highlighted a reasonable hypothesis, in which he stated that using inhibitors which block both ACE2 and ACE pathways in COVID-19 patients could be very beneficial in the treatment of COVID-19. In short, observations from these studies indicate that hypoxia/hypercapnia, a condition that occurs in SARS patients, is highly likely to upregulate the activity of both arms of the RAS. A strong correlation was also observed between the gene expression of ACE2 and that of ACE [117]. Another observation suggested the possibility of a positive feedback induced by SARS-CoV infection, leading to the surface expression of both ACE and ACE2 [118,119,120]. Altogether, these observations indicate that RAS-mediated positive feedback loops can be induced by SARS-CoV-2 at different organ levels. Consequently, in order to block these feedback loops, Zamai, 2020 [116] suggested that different compounds can be produced to inhibit RAS pathways and subsequently to prevent critical, advanced, and untreatable stages of the COVID-19 disease.

IPP, IIAE, LIVTQ, and LVYPFP, which are bioactive peptides derived from whey proteins, were initially characterized as ACE inhibitors through in vitro and in silico assays in our previous works [67,68]. Findings from the current study demonstrate additional novel effects for these bioactive whey-derived peptides as potential ACE2 inhibitors. These results strongly support our hypothesis that these whey-derived peptides not only could exhibit ACE inhibitory activity but also could bind to ACE2 and, as such, could have a potential effect of intervening in the interaction between the ACE2 and SARS-CoV-2 S proteins. Additionally, compared to synthetic ACE-inhibitory drugs, these peptides are from a natural source and do not exhibit toxic side effects, which might also help to reduce the risks associated with traditional drugs in the treatment of COVID-19 infection.

## 3. Materials and Methods

### 3.1. Structure Similarity between 1RL4 and 6M0J

EMBL-EBI (https://www.ebi.ac.uk/, accessed on 14 October 2021) was queried for 1RL4, 6M0J and 2AJF amino acid sequences, together with known three-dimensional (3D) protein structures. Reviewed sequences were selected and the protein sequence files were downloaded. The three sequences were then uploaded to Emboss Needle (https://www.ebi.ac.uk/Tools/psa/emboss_needle/, accessed on 14 October 2021) for pairwise sequence alignment and comparison.

### 3.2. Docking Procedure

**Figure 7:**
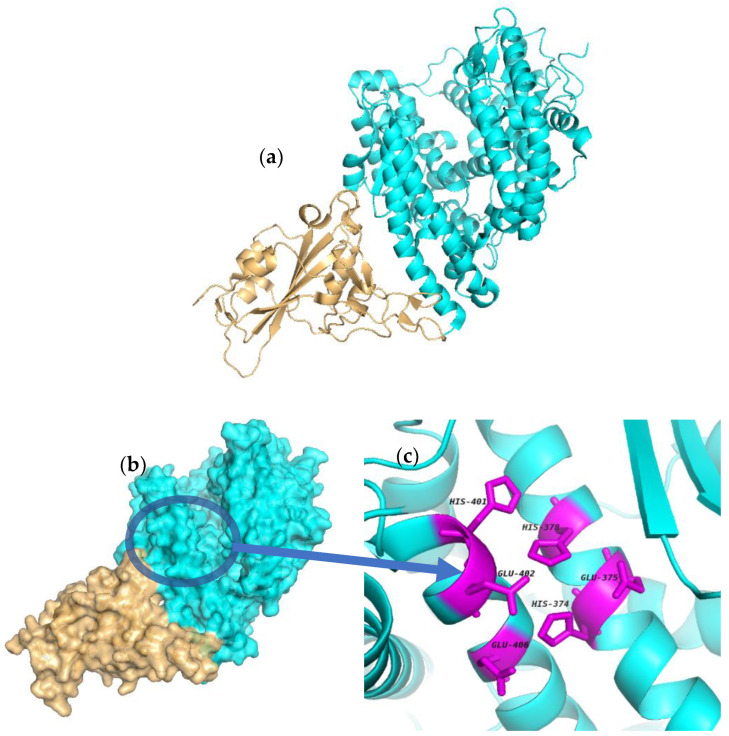
Structure of the SARS-CoV-2 receptor-binding domain (RBD) complexed with ACE2. (**a**) Crystal structure of the SARS-CoV-2 RBD complexed with ACE2. ACE2 is shown in cyan. RBD of SARS-CoV-2 is shown in gold. (**b**) Surface representation of protein 6M0J showing the binding site cleft. (**c**) Amino acid residues in the active site of ACE2 as highlighted in the literature [69,70,71,72,73]. The figures were generated using the software PyMol (Version 2.5.2.)

The X-ray crystallographic structure of ACE2 bound with the CoV-2 S protein (PDB code 6M0J, 2.45 Å resolution), retrieved from the Protein Data Bank (PDB), was chosen as the target protein for the docking studies based on its high-resolution structure co-crystallized with the SARS-CoV-2 S protein. In this crystal structure, the interaction between the SARS-CoV-2 S protein and cell receptor ACE2 is mediated by a defined receptor-binding domain (RBD; Figure 7a) [121]. The binding site cleft of ACE2 (Figure 7b) and details of the active site residues (Figure 7c) have been previously characterized [69,70,71,72,73] and were used in this work to guide the docking procedure.

Whey protein-derived peptides IPP, IIAE, LIVTQ, and LVYPFP were employed as ligands in separate docking runs. The docking methodology has been validated, as previously described, and docking was performed following the same methodology used in our previous work [68] using the docking algorithm Surflex-Dock, as supplied by SYBYL-X 2.1.

To prepare the protein structure for docking, the Biopolymer Structure Preparation Tool, with the implemented default settings provided in the SYBYL program suite, was used. Hydrogens were added to the protein structure in idealized geometries; backbone and sidechains were repaired; residues were protonated; sidechain amides and sidechain bumps were fixed; stage minimization was performed; and all water and any ligand molecules were removed. Using the “Build Protein” tool provided in Sybyl-X, the three-dimensional (3D) structure of each ligand was constructed. Once formed, charges were assigned to each atom of each ligand using Merck Molecular Force Field (MMFF94) charges. Localized energy minimizations were then performed and the final structure for each ligand in its lowest energy conformation was used for successive docking runs. The resulting 3D coordinate files were converted to a MOL2 format for subsequent use in Surflex-Dock experiments, as provided in the SYBYL-X 2.0 software suite.

Surflex-Dock is a search algorithm that employs an empirically derived scoring function whose parameters are based on protein–ligand complexes of established structures and affinities. This procedure uses a “protomol”, which is an idealized active site, as a target to produce presumed poses of molecules. The protomol is utilized as a mimic of the ideal interactions made by a perfect ligand to the active site of the protein. This alignment is based on molecular similarity, which allows for the optimization of theoretically favorable molecular interactions, such as those specified by hydrogen bonds and van der Waals forces. To create a protomol that effectively depicted the binding pocket of interest, the protomol was defined by optimizing the bloat values and threshold to 0 and 0.5, respectively. The bloat values and threshold are two parameters that control the extent of the protomol and its degree of coverage of an active site. The threshold parameter shows the amount of buriedness for the primary volume used to generate the protomol and the bloat value indicates the number of Ångströms by which the search grid beyond that volume should be expanded. In general, it is better to err on the side of a small protomol than on the side of a protomol that is too large [122]. All other parameters within the docking suite were left as the default values, as determined by the software [123,124]. Using the “Docking Suite” application, provided in the SYBYL program suite, each peptide was then individually docked into the protomol site. For further visualization and analysis, Maestro (Schrödinger Release 2021-2: Maestro, Schrödinger, LLC, New York, NY, USA, 2021) was used for the characterization and identification of the hydrogen bonds and salt bridge interactions established between the peptides and residues at the ACE2 active site. Figures were also generated using the software Maestro (Figure 2, Figure 3, Figure 4, Figure 5 and Figure 6).

### 3.3. Docking Validation

In order to validate the accuracy and reliability of the docking procedure to be used in this study, the original ligand (extracted from the coordinate files and taken from the Protein Data Bank; PDB code 1R4L) was docked into the corresponding crystal structure of the receptor using the automated docking procedure in the program Surflex-Dock (SFXC) [125], as provided by SYBYL-X2.1 [69]. The docked ligand mode and orientation from the docking procedure were compared to that found in the actual crystal structure of the complex using PyMol (Version 2.5.2) and PDBeFold (https://www.ebi.ac.uk/msd-srv/ssm/, accessed on 14 October 2021) [126,127]. Following the docking procedure, the RMSD value between the docked ligand and other ligand, as found in the crystal structure, was calculated. The success of the docking process depended upon whether the value of RMSD between the real and best-scored docked conformations was within the 2 Å grid spacing used in the docking procedure [128], and whether the molecular interactions were replicated. In this case, MLN-4760 was docked into the human ACE2 receptor as validation of the docking procedure.

For further validation, the ligand Carnosine, the best-known drug candidate to match an ACE2 inhibitor structure, was docked into the known binding site of ACE2 (PDB code 6M0J) according to the methodology applied by Saadah et al. [40]. The docking results of Carnosine were then compared to the published docking results of the ligand into the active site of ACE2 (PDB code 2AJF) [40]. The ligand Captopril was also prepared and docked into the binding site of ACE2 according to the same methodology.

## 4. Conclusions

For the first time, as presented herein, potential interactions between the naturally produced peptides from whey proteins and ACE2, which is the host cell receptor of SARS-CoV-2, have been examined using a molecular docking approach. Peptides IPP, IIAE, LIVTQ, and LVYPFP all formed strong hydrogen bonds and salt bridge interactions with key residues in the active site of human ACE2. Among the four peptides examined, IPP and IIAE were the most promising candidates to exert an antiviral activity on SARS-CoV-2 through inhibiting ACE2 via specific molecular interactions with key residues of ACE2. IIAE and IPP also formed strong interactions at the active site of ACE2, in common with known potent pharmaceutical ACE2 inhibitors. According to the results of this study, whey-derived peptides IPP, IIAE, LIVTQ, and LVYPFP are suggested as potential candidates to be used in the treatment of SARS-CoV-2 via inhibition of the host cell receptor ACE2. Moreover, in comparison with well-known ACE inhibitory drugs such as Captopril and Lisinopril, the natural peptides produced from whey proteins have a dual inhibitory action against both ACE and ACE2, and may be associated with fewer side-effects, which may represent advantages in the treatment of COVID-19.

The structural insights provided by this molecular docking study are valuable in understanding and manipulating the regulation of ACE2. These peptides could also provide important scaffolds for further insight into the design of novel potent therapeutic inhibitors against SARS-CoV-2 based on ACE2 inhibition. Further in vitro and in vivo studies, however, are needed to further substantiate these whey-derived peptides’ underlying inhibitory mechanisms against ACE2. It also remains unknown whether inhibiting ACE2 would be efficient in attenuating infections by SARS-CoV-2 and, in a similar manner, further research is urgently needed to understand the underlying molecular mechanisms related to these inhibitory mechanisms.

## Figures and Tables

**Table 1:** Docking results of IPP, IIAE, LIVTQ, and LVYPFP.

Protein 6MOJ		Ligand IPP	
Residue	Atom Name	Interaction Type	Distance (Å)
NH1 Arg 273	O− (Pro2)	Salt bridge	3.0
OE1 Glu 375	NH3+ (Ile)	Salt bridge	4.1
OE2 Glu 375	NH3+ (Ile)	Hydrogen bond	2.0
NE2 His 378	NH3+ (Ile)	Hydrogen bond	2.4
OE1 Glu 402	NH3+ (Ile)	Hydrogen bond	2.9
OE2 Glu 402	NH3+ (Ile)	Salt bridge	3.1
		**Ligand IIAE**	
NH2 Arg 273	O1 (Glu)	Salt bridge	3.0
NH2 Arg 273	OE1 (Glu)	Hydrogen bond	2.9
NH1 Arg 273	OE2 (Glu)	Salt bridge	3.0
OE1 Glu 375	NH3+ (Ile)	Salt bridge	3.0
OE2 Glu 375	NH3+ (Ile)	Hydrogen bond	2.6
OE2 Glu 375	NH (Ile)	Hydrogen bond	2.4
OE1 Glu 402	NH3+ (Ile)	Hydrogen bond	2.9
OE2 Glu 402	NH (Ala)	Hydrogen bond	2.9
CG Glu 402	NH3+ (Ile)	Salt bridge	4.3
		**Ligand LIVTQ**	
ND1 His 374	O (Gln)	Hydrogen bond	2.8
OE1 Glu 375	NH3+ (Leu)	Salt bridge	3.5
OE2 Glu 375	NH3+ (Leu)	Hydrogen bond	2.9
OE2 Glu 375	NH (Ile)	Hydrogen bond	2.3
OE1 Glu 402	NH3+ (Leu)	Hydrogen bond	2.0
OE2 Glu 402	NH3+ (Leu)	Salt bridge	4.3
OE2 Glu 402	NH (Val)	Hydrogen bond	2.6
OE1 Glu 406	OH (Thr)	Hydrogen bond	2.9
OE1 Glu 406	NH2 (Gln)	Hydrogen bond	2.9
OE1 Glu 406	NH (Gln)	Hydrogen bond	2.9
OE2 Glu 406	NH (Thr)	Hydrogen bond	2.9
OE2 Glu 406	NH (Val)	Hydrogen bond	2.3
NE2 Gln 442	O (Gln)	Hydrogen bond	2.8
NH2 Arg 518	OH (Thr)	Hydrogen bond	2.1
		**Ligand LVYPFP**	
CG2 Thr 276	O− (Pro)	Hydrogen bond	2.6
OE1 Glu 402	NH3+ (Leu)	Salt bridge	3.8
OE2 Glu 402	NH3+ (Leu)	Hydrogen bond	2.9
CO Glu 406	NH3+ (Leu)	Salt bridge	4.0
OE1 Glu 406	NH (Val)	Hydrogen bond	2.8
OE1 Glu 406	NH (Tyr)	Hydrogen bond	2.4
OE2 Glu 406	NH3+ (Leu)	Hydrogen bond	2.8

## Supplementary Materials

The following are available online at https://www.mdpi.com/article/10.3390/ijms222111662/s1.

## References

[1] World Health Organization (WHO) Cardiovascular Diseases (CVDs): Key Facts. https://www.who.int/news-room/factsheets/detail/cardiovascular-diseases-(cvds).

[2] Townsend N.W.J., Bhatnagar P., Wickramasinghe K., Rayner M. (2014). Cardiovascular Disease Statistics, 2014.

[3] Mills K.T., Stefanescu A., He J. (2020). The global epidemiology of hypertension. Nat. Rev. Nephrol..

[4] Borghi C., Force S.T., Rossi F., Force S.T. (2015). Role of the renin-angiotensin-aldosterone system and its pharmacological inhibitors in cardiovascular diseases: Complex and critical issues. High Blood Press. Cardiovasc. Prev..

[5] Chappell M.C. (2016). Biochemical evaluation of the renin-angiotensin system: The good, bad, and absolute?. Am. J. Physiol. Circ. Physiol..

[6] Griendling K., Murphy T.J., Alexander R.W. (1993). Molecular biology of the renin-angiotensin system. Circulation.

[7] Acharya K.R., Sturrock E.D., Riordan J.F., Ehlers M.R.W. (2003). Ace revisited: A new target for structure-based drug design. Nat. Rev. Drug Discov..

[8] Li G.H., Le G.W., Shi Y.H., Shrestha S. (2004). Angiotensin I–converting enzyme inhibitory peptides derived from food proteins and their physiological and pharmacological effects. Nutr. Res..

[9] Wei L., Alhenc-Gelas F., Corvol P., Clauser E. (1991). The two homologous domains of human angiotensin I-converting enzyme are both catalytically active. J. Biol. Chem..

[10] Sturrock E.D., Natesh R., Van Rooyen J.M., Acharya K.R. (2004). Structure of angiotensin I-converting enzyme. Cell. Mol. Life Sci..

[11] Carey R.M., Siragy H.M. (2003). Newly Recognized Components of the Renin-Angiotensin System: Potential Roles in Cardiovascular and Renal Regulation. Endocr. Rev..

[12] Natesh R., Schwager S.L.U., Sturrock E.D., Acharya K.R. (2003). Crystal structure of the human angiotensin-converting enzyme–lisinopril complex. Nat. Cell Biol..

[13] Tzakos A.G., Galanis A.S., Spyroulias G.A., Cordopatis P., Manessi-Zoupa E., Gerothanassis I.P. (2003). Structure–function discrimination of the N-and C-catalytic domains of human angiotensin-converting enzyme: Implications for Cl–activation and peptide hydrolysis mechanisms. Protein Eng..

[14] Cat A.N.D., Touyz R.M. (2011). Cell Signaling of Angiotensin II on Vascular Tone: Novel Mechanisms. Curr. Hypertens. Rep..

[15] Padia S.H., Carey R.M. (2013). AT2 receptors: Beneficial counter-regulatory role in cardiovascular and renal function. Pflügers Archiv-Eur. J. Physiol..

[16] Santos R.A. (2014). Angiotensin-(1–7). Hypertension.

[17] Tikellis C., Thomas M. (2012). Angiotensin-Converting Enzyme 2 (ACE2) Is a Key Modulator of the Renin Angiotensin System in Health and Disease. Int. J. Pept..

[18] Donoghue M., Hsieh F., Baronas E., Godbout K., Gosselin M., Stagliano N., Donovan M., Woolf B., Robison K., Jeyaseelan R. (2000). A Novel Angiotensin-Converting Enzyme–Related Carboxypeptidase (ACE2) Converts Angiotensin I to Angiotensin 1–9. Circ. Res..

[19] Rice G.I., Thomas D.A., Grant P.J., Turner A.J., Hooper N.M. (2004). Evaluation of angiotensin-converting enzyme (ACE), its homologue ACE2 and neprilysin in angiotensin peptide metabolism. Biochem. J..

[20] Vickers C., Hales P., Kaushik V., Dick L., Gavin J., Tang J., Acton S. (2002). Hy-Drolysis of Biological Peptides by Human Angiotensin-Converting Enzyme-Related Carboxypep-Tidase. J. Biol. Chem..

[21] Santos R.A.S., Simoes e Silva A.C.S., Maric C., Silva D.M.R., Machado R.P., de Buhr I., Heringer-Walther S., Pinheiro S.V.B., Lopes M.T., Bader M. (2003). Angiotensin-(1–7) is an endogenous ligand for the G protein-coupled receptor Mas. Proc. Natl. Acad. Sci. USA.

[22] Ferreira A., Santos R., Almeida A. (2002). Angiotensin-(1–7) improves the post-ischemic function in isolated perfused rat hearts. Braz. J. Med. Biol. Res..

[23] D’Ardes D., Boccatonda A., Rossi I., Guagnano M.T., Santilli F., Cipollone F., Bucci M. (2020). COVID-19 and RAS: Unravelling an Unclear Relationship. Int. J. Mol. Sci..

[24] Ferreira A.J., Santos R.A.S. (2005). Cardiovascular actions of angiotensin-(1-7). Braz. J. Med. Biol. Res..

[25] World Health Organization Coronavirus Disease (COVID-19) Outbreak Situation. https://www.who.int/emergencies/diseases/novel-coronavirus-2019.

[26] Coronaviridae Study Group of the International Committee on Taxonomy of Viruses (2020). The Species Severe Acute Respiratory Syndrome-Related Coronavirus: Classifying 2019-NCoV and Naming It SARS-CoV-2. Nat. Microbiol..

[27] Cui J., Li F., Shi Z.-L. (2019). Origin and evolution of pathogenic coronaviruses. Nat. Rev. Microbiol..

[28] Zhu N., Zhang D., Wang W., Li X., Yang B., Song J., Zhao X., Huang B., Shi W., Lu R. (2020). A Novel Coronavirus from Patients with Pneumonia in China, 2019. N. Engl. J. Med..

[29] Lu R., Zhao X., Li J., Niu P., Yang B., Wu H., Bi Y. (2020). Genomic Characterisa-Tion and Epidemiology of 2019 Novel Coronavirus: Implications for Virus Origins and Receptor Binding. Lancet.

[30] Wei Q., Wang Y., Ma J., Han J., Jiang M., Zhao L., Ye F., Song J., Liu B., National Pathogen Resource Center, Chinese Center for Disease Control and Prevention (2020). Description of the First Strain of 2019-NCoV, C-Tan-NCoV Wuhan Strain—National Pathogen Resource Center, China, 2020. China CDC Wkly..

[31] Wu F., Zhao S., Yu B., Chen Y.-M., Wang W., Hu Y., Song Z.-G., Tao Z.-W., Tian J.-H., Pei Y.-Y. (2020). Complete genome characterisation of a novel coronavirus associated with severe human respiratory disease in Wuhan, China. bioRxiv.

[32] Rothan H.A., Byrareddy S.N. (2020). The epidemiology and pathogenesis of coronavirus disease (COVID-19) outbreak. J. Autoimmun..

[33] Tortorici M.A., Veesler D. (2019). Structural Insights into Coronavirus Entry.

[34] Wrapp D., Wang N., Corbett K.S., Goldsmith J.A., Hsieh C.L., Abiona O., McLel-lan J.S. (2020). Cryo-EM Structure of the 2019-NCoV Spike in the Prefusion Confor-Mation. Science.

[35] Lamers M.M., Beumer J., van der Vaart J., Knoops K., Puschhof J., Breugem T.I., Ravelli R.B.G., Paul van Schayck J., Mykytyn A.Z., Duimel H.Q. (2020). SARS-CoV-2 Productively Infects Human Gut Enterocytes. Science.

[36] Perico L., Benigni A., Remuzzi G. (2020). Should COVID-19 Concern Nephrologists? Why and to What Extent? The Emerging Impasse of Angiotensin Blockade. Nephron.

[37] Zhou F., Yu T., Du R., Fan G., Liu Y., Liu Z., Xiang J., Wang Y., Song B., Gu X. (2020). Clinical course and risk factors for mortality of adult inpatients with COVID-19 in Wuhan, China: A retrospective cohort study. Lancet.

[38] Hoffmann M., Kleine-Weber H., Schroeder S., Krüger N., Herrler T., Erichsen S., Schiergens T.S., Herrler G., Wu N.-H., Nitsche A. (2020). SARS-CoV-2 Cell Entry Depends on ACE2 and TMPRSS2 and Is Blocked by a Clinically Proven Protease Inhibitor. Cell.

[39] Sriram K., Insel P.A. (2020). A hypothesis for pathobiology and treatment of COVID-19: The centrality of ACE1 / ACE2 imbalance. Br. J. Pharmacol..

[40] Saadah L.M., Abu Deiab G.I., Al-Balas Q., Basheti I.A. (2020). Carnosine to Combat Novel Coronavirus (nCoV): Molecular Docking and Modeling to Cocrystallized Host Angiotensin-Converting Enzyme 2 (ACE2) and Viral Spike Protein. Molecules.

[41] Souza P.F., Lopes F.E., Amaral J.L., Freitas C.D., Oliveira J.T. (2020). A molecular docking study revealed that synthetic peptides induced conformational changes in the structure of SARS-CoV-2 spike glycoprotein, disrupting the interaction with human ACE2 receptor. Int. J. Biol. Macromol..

[42] Srivastava N., Garg P., Srivastava P., Seth P.K. (2021). A Molecular Dynamics Simula-Tion Study of the ACE2 Receptor with Screened Natural Inhibitors to Identify Novel Drug Candi-Date against COVID-19. PeerJ.

[43] Ocaranza M.P., Godoy I., Jalil J.E., Varas M., Collantes P., Pinto M., Roman M., Ramirez C., Copaja M., Diaz-Araya G. (2006). Enalapril Attenuates Downregulation of Angiotensin-Converting Enzyme 2 in the Late Phase of Ventricular Dysfunction in Myocardial Infarcted Rat. Hypertension.

[44] Peiris J.S.M., Yuen K.Y., Osterhaus A.D.M.E., Stöhr K. (2003). The Severe Acute Respiratory Syndrome. N. Engl. J. Med..

[45] Kuba K., Imai Y., Rao S., Gao H., Guo F., Guan B., Huan Y., Yang P., Zhang Y., Deng W. (2005). A crucial role of angiotensin converting enzyme 2 (ACE2) in SARS coronavirus–induced lung injury. Nat. Med..

[46] Sun M.L., Yang J.M., Sun Y.P., Su G. (2020). Inhibitors of RAS might be a good choice for the therapy of COVID-19 pneumonia. Chin. J. Tuberc. Respir. Dis..

[47] Vuille-dit-Bille R.N., Camargo S.M., Emmenegger L., Sasse T., Kummer E., Jando J., Hamie Q.M., Meier C.F., Hunziker S., Forras-Kaufmann Z. (2015). Human Intestine Luminal ACE2 and Amino Acid Trans-Porter Expression Increased by ACE-Inhibitors. Amino Acids.

[48] Zheng Y.-Y., Ma Y.-T., Zhang J.-Y., Xie X. (2020). COVID-19 and the cardiovascular system. Nat. Rev. Cardiol..

[49] Kuster G.M., Pfister O., Burkard T., Zhou Q., Twerenbold R., Haaf P., Widmer A.F., Osswald S. (2020). SARS-CoV2: Should Inhibitors of the Renin-Angiotensin System Be Withdrawn in Patients with COVID-19?. Eur. Heart J..

[50] Tomasoni D., Italia L., Adamo M., Inciardi R.M., Lombardi C.M., Solomon S.D., Metra M. (2020). COVID- 19 and heart failure: From infection to inflammation and angiotensin II stimulation. Searching for evidence from a new disease. Eur. J. Heart Fail..

[51] Ferrario C.M., Jessup J., Chappell M.C., Averill D.B., Brosnihan K.B., Tallant E.A., Diz D.I., Gallagher P.E. (2005). Effect of Angiotensin-Converting Enzyme Inhibition and Angiotensin II Receptor Blockers on Cardiac Angiotensin-Converting Enzyme 2. Circulation.

[52] Fang L., Karakiulakis G., Roth M. (2020). Are Patients with Hypertension and Diabetes Mellitus at Increased Risk for COVID-19 Infection?. Lancet Respir. Med..

[53] South A.M., Diz D.I., Chappell M.C. (2020). COVID-19, ACE2, and the cardiovascular consequences. Am. J. Physiol. Circ. Physiol..

[54] Zambelli V., Bellani G., Borsa R., Pozzi F., Grassi A., Scanziani M., Castiglioni V., Masson S., Decio A., Laffey J.G. (2015). Angiotensin-(1–7) improves oxygenation, while reducing cellular infiltrate and fibrosis in experimental Acute Respiratory Distress Syndrome. Intensiv. Care Med. Exp..

[55] Mehta N., Kalra A., Nowacki A.S., Anjewierden S., Han Z., Bhat P., Carmona-Rubio A.E., Jacob M., Procop G.W., Harrington S. (2020). Association of use of angiotensin-converting enzyme inhibitors and angiotensin IIreceptor blockers with testing positive for coronavirus disease 2019 (COVID-19). JAMA Cardiol..

[56] Zhang P., Zhu L., Cai J., Lei F., Qin J.-J., Xie J., Liu Y.-M., Zhao Y.-C., Huang X., Lin L. (2020). Association of Inpatient Use of Angiotensin-Converting Enzyme Inhibitors and Angiotensin II Receptor Blockers With Mortality Among Patients With Hypertension Hospitalized With COVID-19. Circ. Res..

[57] Hippisley-Cox J., Young D., Coupland C., Channon K.M., Tan P.S., A Harrison D., Rowan K., Aveyard P., Pavord I.D., Watkinson P.J. (2020). Risk of severe COVID-19 disease with ACE inhibitors and angiotensin receptor blockers: Cohort study including 8.3 million people. Heart.

[58] Wang G., Yang M.L., Duan Z.L., Liu F.L., Jin L., Long C.B., Lai R. (2021). Dalbavancin Binds ACE2 to Block Its Interaction with SARS-CoV-2 Spike Protein and Is Effec-Tive in Inhibiting SARS-CoV-2 Infection in Animal Models. Cell Res..

[59] Mouffouk C., Mouffouk S., Mouffouk S., Hambaba L., Haba H. (2021). Flavonols as Potential Antiviral Drugs Targeting SARS-CoV-2 Proteases (3CLpro and PLpro), Spike Protein, RNA-Dependent RNA Polymerase (RdRp) and Angiotensin-Converting Enzyme II Receptor (ACE2). Eur. J. Pharmacol..

[60] Brauer F., Schmidt K., Zahn R.C., Richter C., Radeke H.H., Schmitz J.E., Egerer L. (2013). A Rationally Engineered Anti-HIV Peptide Fusion Inhibitor with Greatly Reduced Immu-Nogenicity. Antimicrob. Agents Chemother..

[61] Schütz D., Ruiz-Blanco Y.B., Münch J., Kirchhoff F., Sanchez-Garcia E., Müller J.A. (2020). Peptide and Peptide-Based Inhibitors of SARS-CoV-2 Entry. Adv. Drug Deliv. Rev..

[62] Han D.P., Penn-Nicholson A., Cho M.W. (2006). Identification of Critical Determinants on ACE2 for SARS-CoV Entry and Development of a Potent Entry Inhibitor. Virology.

[63] Martínez-Maqueda D., Miralles B., Recio I., Hernández-Ledesma B. (2012). Antihypertensive peptides from food proteins: A review. Food Funct..

[64] Giromini C., Fekete Á.A., Givens D.I., Baldi A., Lovegrove J.A. (2017). Short-Communication: A Comparison of the In Vitro Angiotensin-1-Converting Enzyme Inhibitory Capacity of Dairy and Plant Protein Supplements. Nutrients.

[65] Fekete A.A., Giromini C., Chatzidiakou Y., Givens D.I., Lovegrove J.A. (2016). Whey Protein Lowers Blood Pressure and Improves Endothelial Function and Lipid Biomarkers in Adults with Prehypertension and Mild Hypertension: Results from the Chronic Whey2Go Ran-Domized Controlled Trial. Am. J. Clin. Nutr..

[66] Fekete A.A., Givens D.I., Lovegrove J.A. (2013). The Impact of Milk Proteins and Pep-Tides on Blood Pressure and Vascular Function: A Review of Evidence from Human Intervention Studies. Nutr. Res. Rev..

[67] Welderufael F.T., Gibson T., Methven L., Jauregi P. (2012). Chemical characterisation and determination of sensory attributes of hydrolysates produced by enzymatic hydrolysis of whey proteins following a novel integrative process. Food Chem..

[68] Chamata Y., Watson K.A., Jauregi P. (2020). Whey-Derived Peptides Interactions with ACE by Molecular Docking as a Potential Predictive Tool of Natural ACE Inhibitors. Int. J. Mol. Sci..

[69] Towler P., Staker B., Prasad S.G., Menon S., Tang J., Parsons T., Ryan D., Fisher M., Williams D., Dales N.A. (2004). ACE2 X-Ray Structures Reveal a Large Hinge-bending Motion Important for Inhibitor Binding and Catalysis. J. Biol. Chem..

[70] Guy J.L., Jackson R.M., Acharya K.R., Sturrock E.D., Hooper A.N.M., Turner A.J. (2003). Angiotensin-Converting Enzyme-2 (ACE2): Comparative Modeling of the Active Site, Specificity Requirements, and Chloride Dependence. Biochemistry.

[71] Teralı K., Baddal B., Gülcan H.O. (2020). Prioritizing potential ACE2 inhibitors in the COVID-19 pandemic: Insights from a molecular mechanics-assisted structure-based virtual screening experiment. J. Mol. Graph. Model..

[72] Guy J.L., Jackson R.M., Jensen H.A., Hooper N., Turner A.J. (2005). Identification of critical active-site residues in angiotensin-converting enzyme-2 (ACE2) by site-directed mutagenesis. FEBS J..

[73] Upreti S., Prusty J.S., Pandey S.C., Kumar A., Samant M. (2021). Identification of novel inhibitors of angiotensin-converting enzyme 2 (ACE-2) receptor from Urtica dioica to combat coronavirus disease 2019 (COVID-19). Mol. Divers..

[74] Nakamura Y., Yamamoto N., Sakai K., Okubo A., Yamazaki S., Takano T. (1995). Purification and Characterization of Angiotensin I-Converting Enzyme Inhibitors from Sour Milk. J. Dairy Sci..

[75] Bimonte S., Crispo A., Amore A., Celentano E., Cuomo A., Cascella M. (2020). Potential Antiviral Drugs for SARS-Cov-2 Treatment: Preclinical Findings and Ongoing Clinical Research. In Vivo.

[76] Centers for Disease Control and Protection, Largest CDC COVID-19 Vaccine Effectiveness Study in Health Workers Shows mRNA Vaccines 94% Effective. https://www.cdc.gov/media/releases/2021/p0514-covid-19-vaccine-effectiveness.htmlhttps://www.cdc.gov/media/releases/2021/p0514-covid-19-vaccine-effectiveness.html.

[77] Dror A.A., Eisenbach N., Taiber S., Morozov N.G., Mizrachi M., Zigron A., Srouji S., Sela E. (2020). Vaccine hesitancy: The next challenge in the fight against COVID-19. Eur. J. Epidemiol..

[78] Arabi Y.M., Alothman A., Balkhy H.H., Al-Dawood A., Aljohani S., Al Harbi S., Kojan S., Aljeraisy M., Deeb A.M., Assiri A.M. (2018). Treatment of Middle East Respiratory Syndrome with a combination of lopinavir-ritonavir and interferon-β1b (MIRACLE trial): Study protocol for a randomized controlled trial. Trials.

[79] COVID-19 Mythbusters—World Health Organization. https://www.who.int/emergencies/diseases/novel-coronavirus-2019/advice-for-public/myth-busters.

[80] Mohanty D., Mohapatra S., Misra S., Sahu P. (2016). Milk derived bioactive peptides and their impact on human health—A review. Saudi J. Biol. Sci..

[81] Pihlanto-Leppälä A. (2000). Bioactive peptides derived from bovine whey proteins: Opioid and ace-inhibitory peptides. Trends Food Sci. Technol..

[82] Vaduganathan M., Vardeny O., Michel T., McMurray J.J.V., Pfeffer M.A., Solomon S.D. (2020). Renin-Angiotensin-Aldosterone System Inhibitors in Patients with Covid-19. N. Engl. J. Med..

[83] Barochiner J., Martínez R. (2020). Use of Inhibitors of the Renin-Angiotensin System in Hypertensive Patients and COVID-19 Severity: A Systematic Review and Meta-Analysis. J. Clin. Pharm. Ther..

[84] Wu C., Chen X., Cai Y., Xia J., Zhou X., Xu S., Huang H., Zhang L., Zhou X., Du C. (2020). Risk Factors Associated With Acute Respiratory Distress Syndrome and Death in Patients With Coronavirus Disease 2019 Pneumonia in Wuhan, China. JAMA Intern. Med..

[85] Sommerstein R., Kochen M.M., Messerli F.H., Gräni C. (2020). Coronavirus Disease 2019 (COVID-19): Do Angiotensin-Converting Enzyme Inhibitors/Angiotensin Receptor Blockers Have a Biphasic Effect?. J. Am. Heart Assoc..

[86] He X., Han B., Mura M., Xia S., Wang S., Ma T., Liu M., Liu Z. (2007). Angiotensin-converting enzyme inhibitor captopril prevents oleic acid-induced severe acute lung injury in rats. Shock.

[87] Danser A.J., Epstein M., Batlle D. (2020). Renin-Angiotensin System Blockers and the COVID-19 Pandemic: At Present There Is No Evidence to Abandon Renin-Angiotensin System Blockers: At Present There Is No Evidence to Abandon Renin-Angiotensin System Blockers. Hypertension.

[88] Lukkarinen H.P., Laine J., Aho H., Zagariya A., Vidyasagar D., Kääpä P.O. (2005). Angiotensin II Receptor Inhibition Prevents Pneumocyte Apoptosis in Surfactant-Depleted Rat Lungs: Apoptosis in Surfactant Depleted-Lungs. Pediatr. Pulmonol..

[89] Medhora M., Gao F., Jacobs E.R., Moulder J.E. (2012). Radiation Damage to the Lung: Mitigation by Angiotensin-Converting Enzyme (ACE) Inhibitors: Radiation Lung Damage and Mitigation. Respirology.

[90] Cohen E.P., Bedi M., Irving A.A., Jacobs E., Tomic R., Klein J., Lawton C.A., Moulder J.E. (2012). Mitigation of Late Renal and Pulmonary Injury After Hematopoietic Stem Cell Transplantation. Int. J. Radiat. Oncol..

[91] Caldeira D., Alarcão J., Carneiro A.V., Costa J. (2012). Risk of pneumonia associated with use of angiotensin converting enzyme inhibitors and angiotensin receptor blockers: Systematic review and meta-analysis. BMJ.

[92] Mortensen E.M., Nakashima B., Cornell J., Copeland L.A., Pugh M.J., Anzueto A., Good C., Restrepo M.I., Downs J.R., Frei C.R. (2012). Population-Based Study of Statins, Angiotensin II Receptor Blockers, and Angiotensin-Converting Enzyme Inhibitors on Pneumonia-Related Outcomes. Clin. Infect. Dis..

[93] Shrikrishna D., Astin R., Kemp P., Hopkinson N. (2012). Renin–angiotensin system blockade: A novel therapeutic approach in chronic obstructive pulmonary disease. Clin. Sci..

[94] Harder E.M., Park H.S., Nath S.K., Mancini B.R., Decker R.H. (2015). Angiotensin-converting enzyme inhibitors decrease the risk of radiation pneumonitis after stereotactic body radiation therapy. Pr. Radiat. Oncol..

[95] ESH STATEMENT ON COVID-19|European Society of Hypertension. https://www.eshonline.org/esh-content/uploads/2020/06/Statement-ESH-on-Hypertension-RAS-Blockers-and-COVID-19-Update-April-15-2020.pdf.

[96] Position Statement of the ESC Council on Hypertension on ACE-Inhibitors and Angiotensin Receptor Blockers. https://www.sphta.org.pt/files/european_society_of_hypertension_-_statement_on_covid-19.pdf.

[97] Bozkurt B., Kovacs R., Harrington B. (2020). Joint HFSA/ACC/AHA Statement Addresses Concerns Re: Using RAAS Antagonists in COVID-19. J. Card. Fail..

[98] Henry C., Zaizafoun M., Stock E., Ghamande S., Arroliga A.C., White H.D. (2018). Impact of angiotensin-converting enzyme inhibitors and statins on viral pneumonia. Bayl. Univ. Med. Cent. Proc..

[99] Yang G. (2020). Effects of ARBs and ACEIs on Virus Infection, Inflammatory Status and Clinical Outcomes in COVID-19 Patients with Hypertension: A Single Center Retrospective Study. Hypertension.

[100] Meng J., Xiao G., Zhang J., He X., Ou M., Bi J., Yang R., Di W., Wang Z., Li Z. (2020). Renin-angiotensin system inhibitors improve the clinical outcomes of COVID-19 patients with hypertension. Emerg. Microbes Infect..

[101] Gu H., Xie Z., Li T., Zhang S., Lai C., Zhu P., Wang K., Han L., Duan Y., Zhao Z. (2016). Angiotensin-converting enzyme 2 inhibits lung injury induced by respiratory syncytial virus. Sci. Rep..

[102] Li X., Molina-Molina M., Abdul-Hafez A., Uhal V., Xaubet A., Uhal B.D. (2008). Angiotensin converting enzyme-2 is protective but downregulated in human and experimental lung fibrosis. Am. J. Physiol. Cell. Mol. Physiol..

[103] Liu Y., Yang Y., Zhang C., Huang F., Wang F., Yuan J., Wang Z., Li J., Li J., Feng C. (2020). Clinical and biochemical indexes from 2019-nCoV infected patients linked to viral loads and lung injury. Sci. China Life Sci..

[104] Oudit G.Y., Kassiri Z., Jiang C., Liu P.P., Poutanen S., Penninger J., Butany J. (2009). SARS-coronavirus modulation of myocardial ACE2 expression and inflammation in patients with SARS. Eur. J. Clin. Investig..

[105] Watkins J. (2020). Preventing a covid-19 pandemic. BMJ.

[106] Ishiyama Y., Gallagher P.E., Averill D.B., Tallant E.A., Brosnihan K.B., Ferrario C.M. (2004). Upregulation of Angiotensin-Converting Enzyme 2 After Myocardial Infarction by Blockade of Angiotensin II Receptors. Hypertension.

[107] Epelman S., Tang W.W., Chen S.Y., Van Lente F., Francis G.S., Sen S. (2008). Detection of Soluble Angiotensin-Converting Enzyme 2 in Heart Failure: Insights Into the Endogenous Counter-Regulatory Pathway of the Renin-Angiotensin-Aldosterone System. J. Am. Coll. Cardiol..

[108] Furuhashi M., Moniwa N., Mita T., Fuseya T., Ishimura S., Ohno K., Shibata S., Tanaka M., Watanabe Y., Akasaka H. (2015). Urinary Angiotensin-Converting Enzyme 2 in Hypertensive Patients May Be Increased by Olmesartan, an Angiotensin II Receptor Blocker. Am. J. Hypertens..

[109] Park J.-H., Oh Y.-S., Kim J.-H., Chung W.-B., Oh S.-S., Lee D.-H., Choi Y.-S., Shin W.-S., Park C.-S., Youn H.-J. (2009). Effect of Angiotensin Converting Enzyme Inhibitors and Angiotensin Receptor Blockers on Patients Following Ablation of Atrial Fibrillation. Korean Circ. J..

[110] Lely A.T., Hamming I., van Goor H., Navis G.J. (2004). Renal ACE2 expression in human kidney disease. J. Pathol..

[111] Burrell L.M., Cooper M.E., Johnston C.I. (2005). Myocardial infarction increases ACE2 expression in rat and humans: Reply. Eur. Heart J..

[112] Hampl V., Herget J., Bíbová J., Baňasová A., Husková Z., Vaňourková Z., Jíchová Š., Kujal P., Vernerová Z., Sadowski J. (2015). Intrapulmonary Activation of the Angiotensin-Converting Enzyme Type 2/Angiotensin 1–7/G-Protein-Coupled Mas Receptor Axis Attenuates Pulmonary Hypertension in Ren-2 Transgenic Rats Exposed to Chronic Hypoxia. Physiol. Res..

[113] Wood C.E., Kane C., Raff H. (1990). Peripheral Chemoreceptor Control of Fetal Renin Re-Sponses to Hypoxia and Hypercapnia. Circ. Res..

[114] Yamaguchi K., Suzuki K., Naoki K., Nishio K., Sato N., Takeshita K., Kudo H., Aoki T., Suzuki Y., Miyata A. (1998). Response of Intra-acinar Pulmonary Microvessels to Hypoxia, Hypercapnic Acidosis, and Isocapnic Acidosis. Circ. Res..

[115] Liao X., Wang L., Yang C., He J., Wang X., Guo R., Lan A., Dong X., Yang Z., Wang H. (2011). Cyclooxygenase Mediates Cardioprotection of Angiotensin-(1–7) against Ische-Mia/Reperfusion-Induced Injury through the Inhibition of Oxidative Stress. Mol. Med. Rep..

[116] Zamai L. (2020). The Yin and Yang of ACE/ACE2 Pathways: The Rationale for the Use of Renin-Angiotensin System Inhibitors in COVID-19 Patients. Cells.

[117] Wakahara S., Konoshita T., Mizuno S., Motomura M., Aoyama C., Makino Y., Kato N., Koni I., Miyamori I. (2007). Synergistic Expression of Angiotensin-Converting Enzyme (ACE) and ACE2 in Human Renal Tissue and Confounding Effects of Hypertension on the ACE to ACE2 Ratio. Endocrinology.

[118] Jia H.P., Look D.C., Tan P., Shi L., Hickey M., Gakhar L., Chappell M.C., Wohlford-Lenane C., McCray P.B. (2009). Ectodomain Shedding of Angiotensin Converting Enzyme 2 in Hu-Man Airway Epithelia. Am. J. Physiol. Cell. Mol. Physiol..

[119] Haga S., Yamamoto N., Nakai-Murakami C., Osawa Y., Tokunaga K., Sata T., Yamamoto N., Sasazuki T., Ishizaka Y. (2008). Modulation of TNF-α-Converting Enzyme by the Spike Protein of SARS-CoV and ACE2 Induces TNF-α Production and Facilitates Viral Entry. Proc. Natl. Acad. Sci. USA.

[120] Clarke N.E., Belyaev N.D., Lambert D.W., Turner A.J. (2013). Epigenetic Regulation of Angioten-Sin-Converting Enzyme 2 (ACE2) by SIRT1 under Conditions of Cell Energy Stress. Clin. Sci..

[121] Lan J., Ge J., Yu J., Shan S., Zhou H., Fan S., Zhang Q., Shi X., Wang Q., Zhang L. (2020). Structure of the SARS-CoV-2 spike receptor-binding domain bound to the ACE2 receptor. Nature.

[122] Sharma R., Dhingra N., Patil S. (2016). CoMFA, CoMSIA, HQSAR and molecular docking analysis of ionone-based chalcone derivatives as antiprostate cancer activity. Indian J. Pharm. Sci..

[123] Ai Y., Wang S.-T., Sun P.-H., Song F.-J. (2011). Combined 3D-QSAR Modeling and Molecular Docking Studies on Pyrrole-Indolin-2-ones as Aurora A Kinase Inhibitors. Int. J. Mol. Sci..

[124] Lan P., Chen W.N., Chen W.M. (2011). Molecular modeling studies on imidazo [4, 5-b] pyridine derivatives as Aurora A kinase inhibitors using 3D-QSAR and docking approaches. Eur. J. Med. Chem..

[125] Jain R.K. (2003). Molecular regulation of vessel maturation. Nat. Med..

[126] Schrodinger LLC (2015). Version 1.8, The PyMOL Molecular Graphics System.

[127] (2019). PDBeFold—Structure Similarity, Embl-Ebi. http://www.ebi.ac.uk/msd-srv/ssm/cgi-bin/ssmserver.

[128] Wang R., Lu Y., Wang S. (2003). Comparative Evaluation of 11 Scoring Functions for Molecular Docking. J. Med. Chem..

