# Supplementary material for: Whey-Derived Peptides at the Heart of the COVID-19 Pandemic
Source: Int J Mol Sci. 2021 Oct 28;22(21):11662. doi: 10.3390/ijms222111662 (PMC8584039; doi:10.3390/ijms222111662)
Supplement: Supplementary file 1 [file ijms-22-11662-s001.zip › ijms-1410209-supplementary.pdf]

Whey-Derived Peptides at the Heart of the COVID-19 Pandemic

Supplementary data

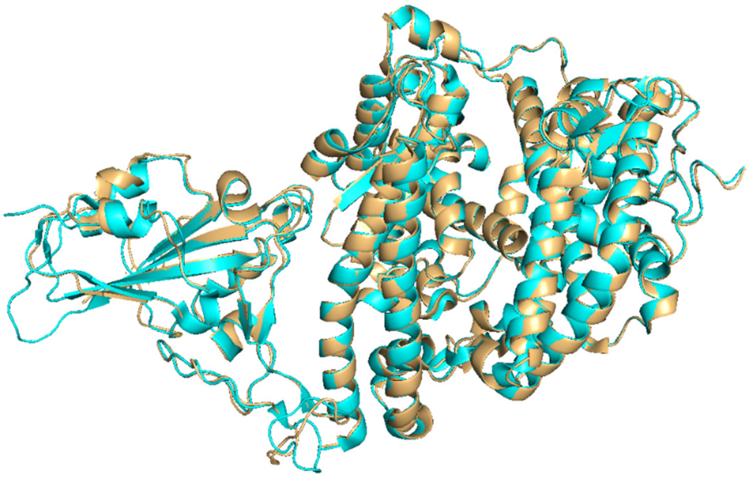

Figure S1. Superimposition of 6M0J and 2AJF crystal structures. RMSD value calculated = 0.46.

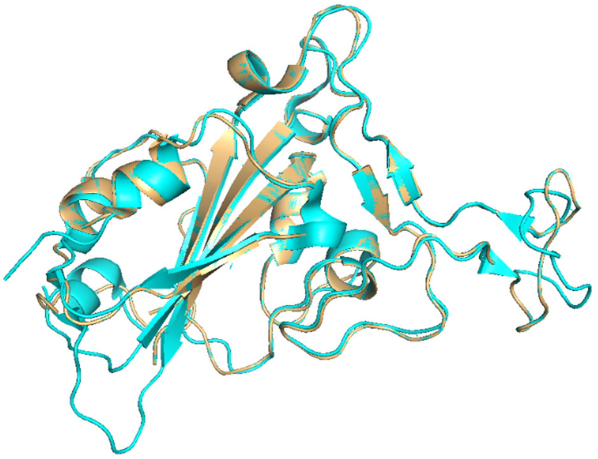

Figure S2. Superimposition of the SARS-CoV 2 S protein receptor-binding domain (Residues 337- 515) of 6M0J and SARS-CoV S protein receptor-binding domain (Residues 323-501) of 2AJF. RMSD value calculated = 0.41.

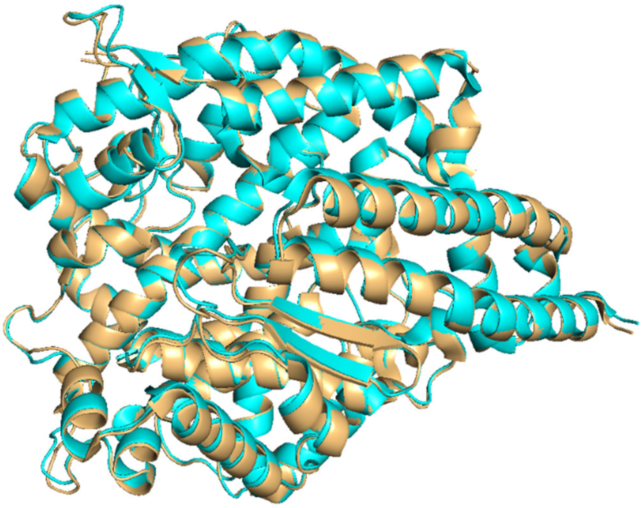

Figure S3. Superimposition of the ACE2 sequence (Residues 21- 615) of 6M0J and the ACE2 sequence (Residues 21- 615) of 2AJF. RMSD value calculated = 0.43.

```
6M0J               1 CPFGEVFNATRFASVYAWNRKRISNCVADYSVLYNSASFSTFKCYGVSPT     50
                     ||||||||||:|.|||||.||:||||||||||||||..||||||||||.|
2AJF               1 CPFGEVFNATKFPSVYAWERKKISNCVADYSVLYNSTFFSTFKCYGVSAT     50

6M0J              51 KLNDLCFTNVYADSFVIRGDEVRQIAPGQTGKIADYNYKLPDDFTGCVIA    100
                     |||||||:||||||||::||:||||||||||.||||||||||||.|||:|
2AJF              51 KLNDLCFSNVYADSFVVKGDDVRQIAPGQTGVIADYNYKLPDDFMGCVLA    100

6M0J             101 WNSNNLDSKVGGNYNYLYRLFRKSNLKPFERDISTEIYQAGSTPCNGVEG    150
                     ||:.|:|:...|||||.||..|...|:|||||||...:.....||. ...
2AJF             101 WNTRNIDATSTGNYNYKYRYLRHGKLRPFERDISNVPFSPDGKPCT-PPA    149

6M0J             151 FNCYFPLQSYGFQPTNGVGYQPYRVVVLSFE    181
                     .|||:||..|||..|.|:|||||||||||||
2AJF             150 LNCYWPLNDYGFYTTTGIGYQPYRVVVLSFE    180
```

(I) residues are identical

(.) conserved change

(:) part of the residue is similar but not that conserved

% similarity = 80.2

% identity = 72.4

Figure S4. EMBOSS NEEDLE pairwise sequence alignment results between SARS CoV-2 S protein receptor-binding site of 6M0J and SARS CoV S protein receptor-binding site of 2AJF.

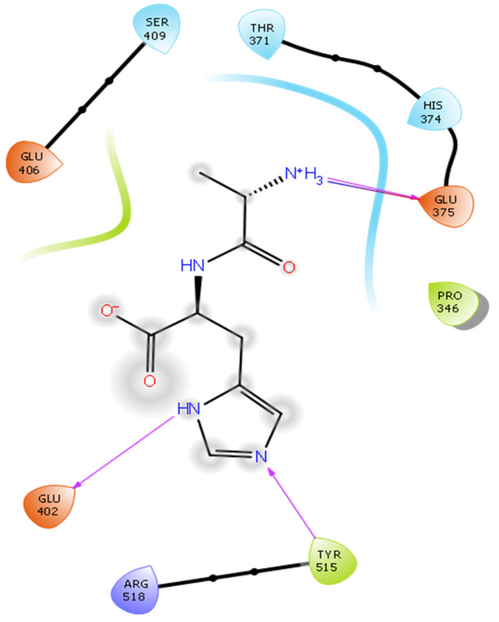

Figure S5. Docking results of the peptide Carnosine in the human angiotensin converting enzyme 2 (ACE2) active site (PDB code 2AJF). The interactions of human ACE2 residues with the peptide (represented in black) are indicated by arrows of different colours with purple representing hydrogen bond interactions, and blue arrows representing salt bridge interactions.

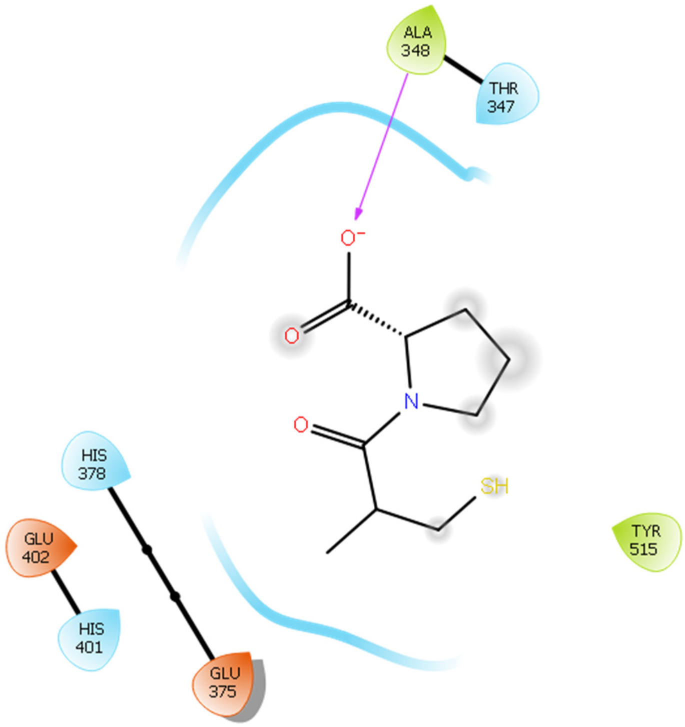

Figure S6. Docking results of Captopril in the human angiotensin converting enzyme 2 (ACE2) active site (PDB code 2AJF). The interaction of human ACE2 residues with Captopril (represented in black) is indicated by a purple arrow representing hydrogen bond interactions.

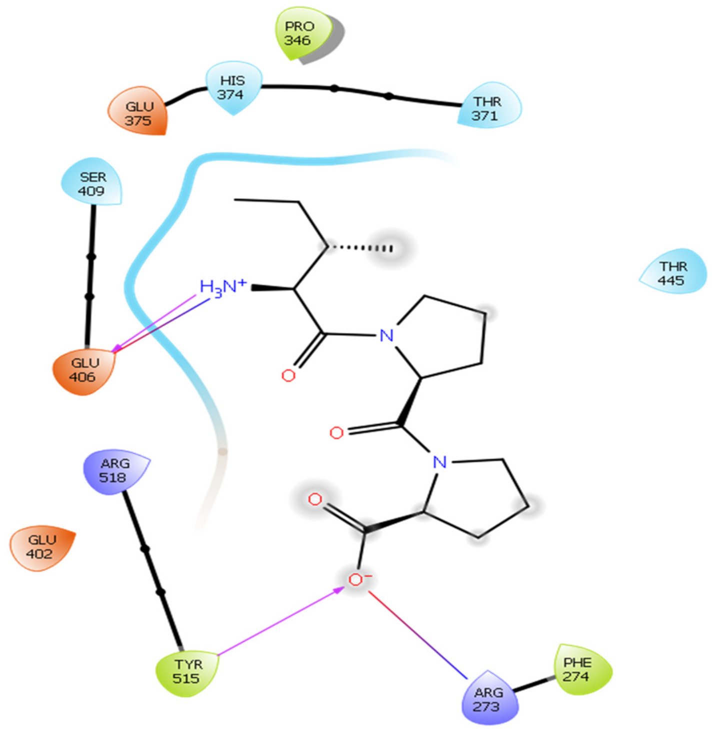

Figure S7. Docking results of the peptide IPP in the active site of human angiotensin 2-converting enzyme (ACE2) (PDB code 2AJF). Interactions of human ACE2 residues with the peptide IPP (represented in black) are indicated by arrows of different colours, with purple representing hydrogen bond interactions and blue arrows representing salt bridge interactions.

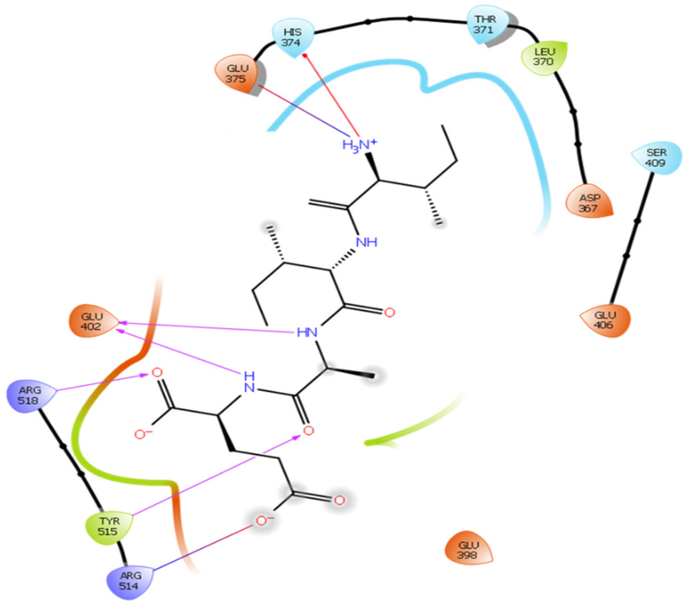

Figure S8. Docking results of the peptide IIAE in the active site human ACE2 (PDB code 2AJF). The interactions of human ACE2 residues with the peptide IIAE (represented in black) are indicated by arrows of different colours with purple representing hydrogen bond interactions, and blue arrows representing salt bridge interactions.

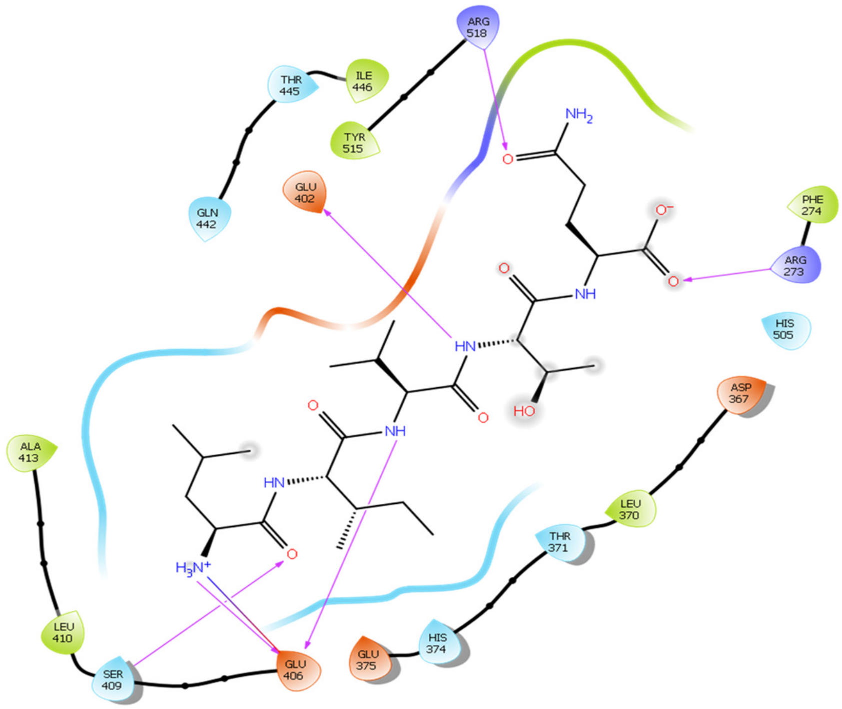

Figure S9. Docking results of the peptide LIVTQ in the human ACE2 active site (PDB code 2AJF). The interactions of human ACE2 residues with the peptide LIVTQ (represented in black) are indicated by arrows of different colours with purple representing hydrogen bond interactions, and blue arrows rep-resenting salt bridge interactions.

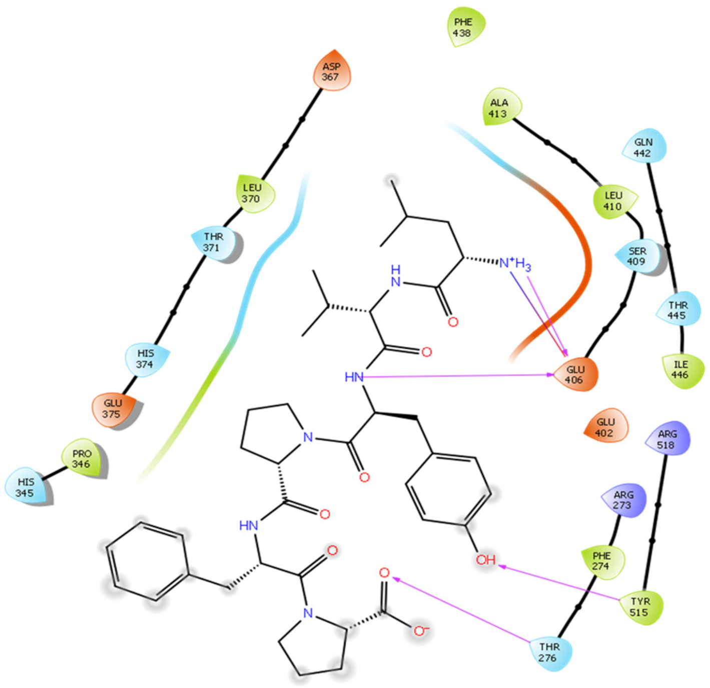

Figure S10. Docking results of the peptide LVYPFP in the human ACE2 active site (PDB code 2AJF). The interactions of human ACE2 residues with the peptide (represented in black) are indicated by arrows of different colours with purple representing hydrogen bond interactions, and blue arrows representing salt bridge interactions.

Table S1. Docking results of IPP, IIAE, LIVTQ, and LVYPFP.

**Protein 2AJF**		**Ligand IPP**	
Residue	Atom Name	Interaction Type	Distance (Å)
OE2 Glu 406	NH3+ (Ile)	Hydrogen bond	2.9
OE2 Glu 406	NH3+ (Ile)	Salt bridge	3.3
NH1 Arg273	O- (Pro)	Salt bridge	4.6
OH Tyr 515	O- (Pro)	Hydrogen bond	3.0
		**Ligand IIAE**	
OH Tyr 515	O (Ala)	Hydrogen bond	2.8
NH2 Arg 518	O2 (Glu)	Hydrogen bond	2.9
OE1 Glu 402	NH (Glu)	Hydrogen bond	3.0
OE1 Glu 402	NH (Ala)	Hydrogen bond	2.7
OE2 Glu 375	NH_3^+ (Ile)	Salt bridge	3.2
NH1 Arg 514	O- (Glu)	Salt bridge	3.7
		**Ligand LIVTQ**	
NH2 Arg273	O- (Gln)	Hydrogen bond	2.9
NH2 Arg 518	O (Gln)	Hydrogen bond	3.0
OE1 Glu 406	NH3+ (Leu)	Hydrogen bond	2.8
OE2 Glu 406	NH (Ile)	Hydrogen bond	2.9
CD Glu 406	NH3+ (Leu)	Salt bridge	3.5
OG Ser 409	O (Leu)	Hydrogen bond	3.0
OE1 Glu 402	NH (Thr)	Hydrogen bond	3.0
		**Ligand LVYPFP**	
OE1 Glu 406	NH3+(Leu)	Hydrogen bond	3.0
OE2 Glu 406	NH (Val)	Hydrogen bond	2.8
CD Glu 406	NH3+ (Leu)	Salt bridge	4.1
OH Tyr 515	OH (Tyr)	Hydrogen bond	2.8
OG1 Thr 276	O (Pro)	Hydrogen bond	2.9
